# CIS5/405: Web Technology in Healthcare - Delivering Electronic Records Using the Clinical Intranet

**DOI:** 10.2196/jmir.1.suppl1.e10

**Published:** 1999-09-19

**Authors:** M Berger

**Affiliations:** ^1^MODCOMP GmbH, KoelnGermany

**Keywords:** Information Management, Electronic Records, Web Technology, Patient Administration System, Host-to-Web

## Abstract

**Introduction:**

The development of electronic records - EPR & EHR (Electronic Patient Records & Electronic Health Records) - requires the use of innovative technology. With the emergence of web enabled applications, that technology is now available. In this paper, we consider the opportunities afforded by web technology and articulate their vision for making electronic records an affordable reality through the use of the ViewMax Integration Server. It is designed to be used as a discussion document for Health Authorities, Primary Care Groups and Trusts when considering their shared strategies for building electronic records.

**Methods:**

Hospitals that have developed, or are developing EPR, have generally adopted one of the following approaches: Big Bang solutions or Interfaced solutions. Whilst both of these models have their merits, both also have significant limitations and disadvantages. With the advent of web technology, a "third way" has emerged. Through the development of e-commerce in the commercial sector, sophisticated Host-to-Web integration tools are now available, providing facilities which would have seemed impossible only a few years ago:

- Systems can now be integrated and accessed through an industry standard web browser.
- Legacy systems can be provided with a modern, intuitive interface, designed to support the needs of particular groups of users, and data from multiple, disparate systems, applications and screens can be combined into a single web page.
- Data from one system can be easily transferred to other applications.
- New applications developed using modern databases can be seamlessly integrated with existing host systems.
- Most importantly, this new approach enables full interactive access to legacy technologies using a browser, without requiring any modification to host systems.

Utilising the latest Web integration tools it is now possible to incrementally develop cost-effective electronic records.

**Results:**

The hospital is an average acute district general hospital, and runs a PAS and the normal range of departmental systems - pathology, radiology, pharmacy, theatres and maternity. The PAS shares demographic details with the departmental systems via point to point interfaces, so that all use the hospital number as the main patient identifier. The hospital wants to make better use of these systems to meet the following requirements:

- Diagnostic result reporting for clinical staff.
- Electronic ordering of pathology and radiology services.
- Simplified ward based access to PAS ADT functions.
- Patient event history.
- Discharge letter production.

**Discussion:**

Web integration tools enable applications to be integrated live within the user interface, and can be used to build "new" applications, by consolidating pieces of functionality from existing and new systems seamlessly within the browser.

